# Comparative Evaluation of the Adaptation Accuracy of Two Commercially-Available Denture Base Resins: An In Vitro Study

**DOI:** 10.7759/cureus.88946

**Published:** 2025-07-28

**Authors:** Pratti N Aneesha, Muvva Suresh Babu, Gottumukkala Vineela, Samyuktha Devarapalli, Navya K Chowdary, Husnara Shaik

**Affiliations:** 1 Department of Prosthodontics, Sibar Institute of Dental Sciences, Guntur, IND

**Keywords:** adaptation, dental materials, denture base, in vitro study, microscope, prosthodontics, thermoplastic resin, visible light-cured resin

## Abstract

Introduction

This in vitro study aimed to evaluate and compare the adaptation accuracy of two commercially available denture base polymers: visible light-cured (VLC) resin and thermoplastic resin. The specific objectives were to assess the adaptation accuracy of each material at different anatomical sites and to determine which material offered a superior fit across the denture-bearing maxillary arch.

Materials and methods

This cross-sectional study was conducted at the Department of Prosthodontics, Sibar Institute of Dental Sciences, Guntur, India, between June and December 2024. Forty standardized edentulous maxillary casts were fabricated using a silicone mold (Zhermack S.p.A., Badia Polesine, Italy) and Type III dental stone (Kalabhai Karson Pvt. Ltd., Mumbai, India). The samples were randomly divided into two groups of 20. Group A (n=20) used VLC resin sheets (Delta VLC, Delta Dental Solutions, Chennai, India), which were adapted to the casts using finger pressure and cured in a light-curing unit (Triad 2000, Dentsply Sirona, Charlotte, North Carolina, USA). Group B (n=20) consisted of thermoplastic denture base sheets (3A Medes, Seoul, South Korea) adapted using a vacuum-forming machine (Biostar; Scheu-Dental GmbH, Iserlohn, Germany) under consistent heat and pressure. All samples were transversely sectioned at the canine, molar, and posterior mid-palatal regions. Adaptation accuracy at nine specific points was measured using a stereomicroscope (Leica M205 C; Leica Microsystems, Wetzlar, Germany) at 20x magnification.

Results

Statistically significant differences were observed both within and between the groups. VLC resins demonstrated the best adaptation in the canine region (mean: 370.37 µm), whereas thermoplastic resins showed superior adaptation in the posterior mid-palatal region (mean: 310.79 µm). Overall, the thermoplastic resins exhibited significantly lower mean discrepancies (430.32 µm) than VLC resins (570.05 µm), with the greatest difference observed in the posterior mid-palatal region (p<0.001).

Conclusion

Thermoplastic denture base materials provided better adaptation accuracy than VLC resins, particularly in the anatomically challenging posterior regions. These findings suggest that thermoplastic resins may offer improved clinical outcomes in cases where a precision fit is essential.

## Introduction

A well-fitted and dimensionally stable denture base is critical for the success of prosthetic restorations, ensuring optimal intraoral retention, stability, and patient comfort [1]. The accuracy of the adaptation of a denture base to the underlying oral tissues is influenced by multiple factors, including the choice of material, fabrication techniques, and processing conditions [2]. Dimensional changes during processing, such as shrinkage or expansion, can compromise the fit, leading to reduced retention and stability, which may affect the functionality and longevity of the dentures [1,2]. Historically, materials such as shellac, thermoform plastics, polycaprolactone, self-cure acrylic resins, and heat-cure acrylic resins have been used to fabricate custom trays, record bases, and dentures [3]. Acrylic resins, particularly polymethyl methacrylate (PMMA), have been widely used because of their versatility and cost-effectiveness [2]. However, acrylic resins have limitations, including polymerization shrinkage, water absorption, and discoloration over time, which can lead to volumetric changes and compromised aesthetics in the oral environment. These dimensional variations are influenced by factors such as curing methods, material thickness, and thermal expansion differences between the resin and the gypsum mold used during fabrication [4,5].

Heat-cured acrylic resins, for instance, undergo polymerization shrinkage during curing, and the release of internal stress during cooling can cause dimensional inaccuracies. These changes are particularly pronounced in areas with varying base thicknesses, leading to inconsistent adaptation to the master cast [6]. Such discrepancies can result in gaps between the denture base and oral tissues, particularly in the posterior palatal region [7]. This gap formation is attributed to the release of internal stress, which causes the denture flange to move inward, elevating the midpalatal zone and creating premature contact with the mold. To address these challenges, alternative materials such as visible light-cured (VLC) resins and thermoplastic materials have emerged as promising substitutes [8]. VLC resins, introduced in 1984, utilize urethane dimethacrylate (UDMA) polymers and offer advantages such as reduced allergic reactions, minimal odor, and elimination of the labor-intensive lost-wax technique associated with conventional PMMA [9]. These materials exhibit improved dimensional stability and are less prone to processing errors than other materials.

In contrast, thermoplastic materials are monomer-free, high-molecular-weight compounds known for their flexibility, biocompatibility, and resistance to liquid absorption. Commonly used thermoplastics include polyamides, acetals, acrylic thermoplastics, polyolefins, and polyesters. Polypropylene is a popular choice because of its semicrystalline structure, transparency, and wear resistance [10]. These properties make thermoplastics suitable for use in temporary dentures, partial dentures, and implant-supported prostheses [1]. However, both VLC and thermoplastic materials face challenges related to processing shrinkage, particularly in the palatal region, which can lead to distortion and affect adaptation accuracy.

This study aimed to evaluate and compare the adaptation accuracy of VLC and thermoplastic denture base resins to determine their effectiveness in producing well-fitting dentures in the short term

## Materials and methods

This cross-sectional in vitro study was conducted at the Department of Prosthodontics, Sibar Institute of Dental Sciences, Guntur, India, between June and December 2024. Ethical approval was obtained from the Institutional Ethical Committee of the Sibar Institute of Dental Sciences and Hospital (Pr.190/IEC/SIBAR/2023). An ethical waiver was granted because the study was conducted in vitro and did not involve human subjects, thereby negating the need for informed consent. This study adhered to the principles outlined in the Declaration of Helsinki for ethical research purposes.

The sample size was determined using G*Power software (version 3.1.9.2, Heinrich-Heine-Universität Düsseldorf, Düsseldorf, Germany) based on a prior study comparing denture material adaptation accuracy with an effect size of 0.34 [11]. With a power of 95%, confidence level of 95%, and alpha error of 5%, the minimum required sample size was calculated to be 20 per group.

Forty samples were equally divided into two groups: Group A (n=20) was fabricated using VLC resin sheets (Delta, Delta Dental Sol., Chennai, India), and Group B (n=20) was fabricated using thermoplastic denture base material (3A Medes, Seoul, South Korea). The eligibility criteria for sample inclusion required the use of a completely edentulous maxillary silicone mold with a well-formed rounded ridge to ensure uniformity. Casts with defects, irregular ridge forms, or incomplete gypsum settings were excluded to maintain consistency during the evaluation.

**Figure 1:**
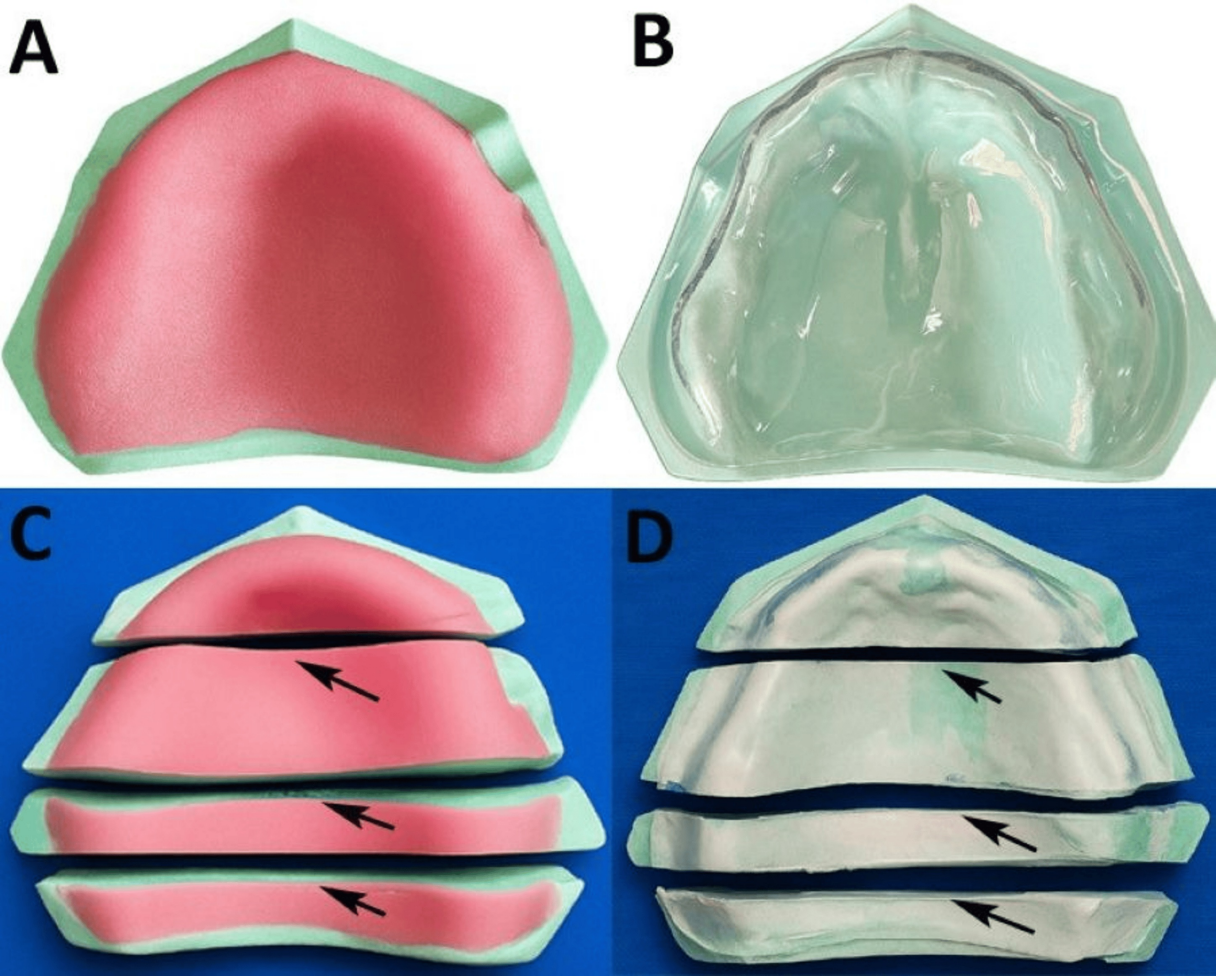
Sample fabrication - Original images of the maxillary casts from the study (A) Visible light-cured (VLC) denture base resin sheets adapted to the maxillary cast. (B) Thermoplastic denture base resin sheet adapted to the maxillary cast. (C) Sectioning of the VLC-adapted maxillary cast at the canine, molar and posterior mid-palatal area (black arrows). (D) Sectioning of thermoplastic-adapted maxillary cast at the canine, molar and posterior mid-palatal area (black arrows).

To fabricate the samples, 40 maxillary casts were prepared using a Type III gypsum product (Kalabhai Karson Pvt. Ltd., Mumbai, India). A silicone rubber mold (Zhermack S.p.A., Badia Polesine, Italy) was used to create a standardized edentulous maxillary cast. Gypsum was mixed with water in a rubber bowl using a spatula, poured into the mold under vibration using a vibrator (Renfert GmbH, Hilzingen, Germany) to eliminate air bubbles, and allowed to set. For Group A, the VLC resin sheets were adapted to the casts using finger pressure, starting from the center and moving toward the periphery to minimize the air entrapment (Figure 1A).

The excess material was trimmed using a precision cutting machine (Buehler, Lake Bluff, Illinois, USA), and the sheets were cured in a light-curing unit (Triad 2000, Dentsply Sirona, Charlotte, North Carolina, USA) for 11 min using the P1 cycle, according to the manufacturer’s instructions (Figure 1A). For Group B, 2 mm thick thermoplastic sheets were heated in a vacuum-forming machine (Biostar, Scheu-Dental GmbH, Iserlohn, Germany) for at least five minutes and adapted to the casts under vacuum for two minutes using hand pressure with gauze to ensure uniform adaptation (Figure 1B).

**Figure 2:**
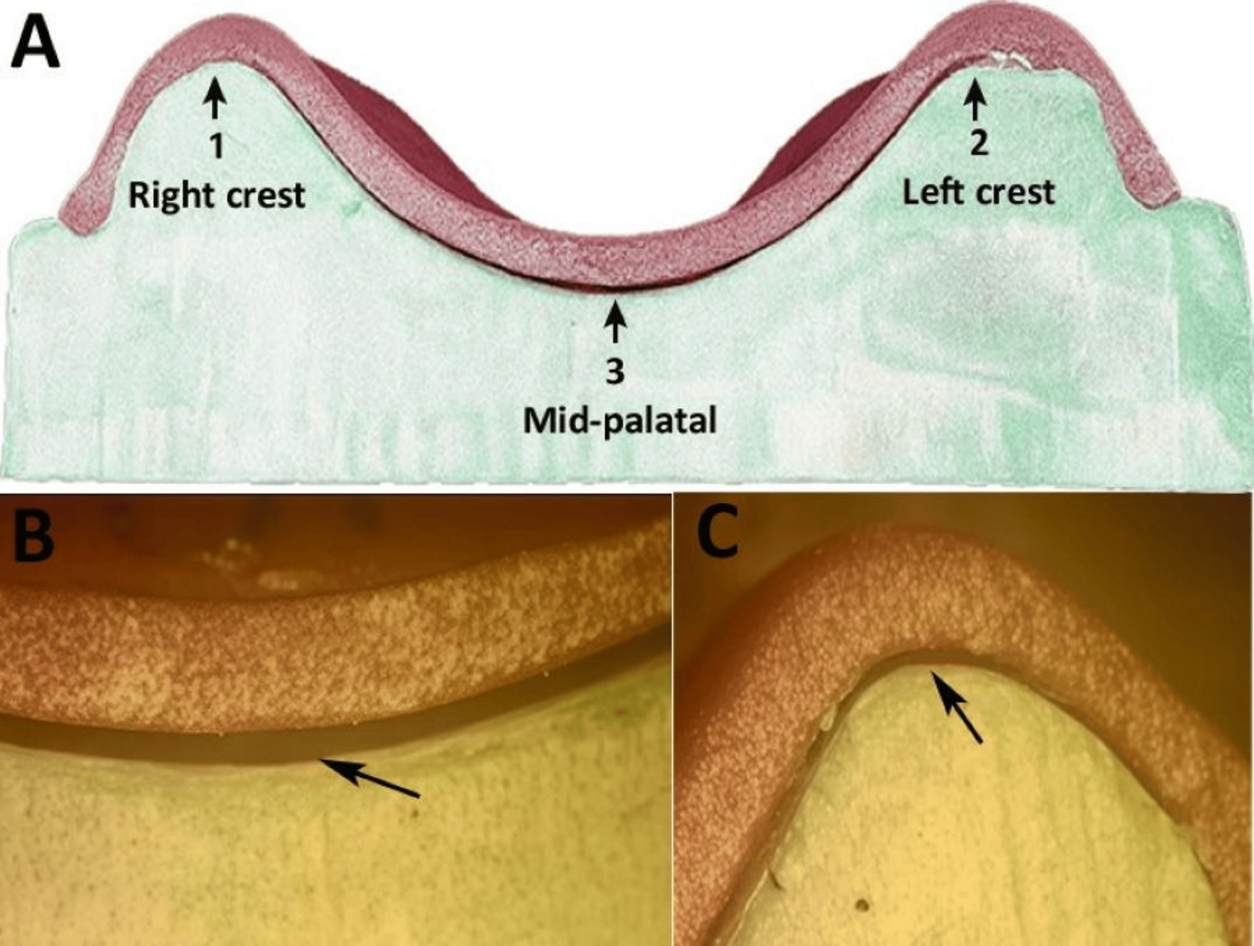
Fixed reference points for discrepancy assessment - original images of the maxillary casts from the study (A) Discrepancy assessment points at the right and left ridge crest and the mid-palatal region (black arrows). (B) Measuring discrepancy in microns at the mid-palatal area (20x magnification by stereomicroscope). (C) Measuring discrepancy in microns at the crestal area (20x magnification by stereomicroscope).

To ensure measurement accuracy and consistency, all the samples were transversely sectioned using a precision cutting machine at predetermined locations: the canine region, molar region, and posterior palatal seal region (Figures 1C, 1D). A standardized reference system was established using fixed anatomical landmarks on the edentulous maxillary casts, including the incisive papilla as the anterior reference and the fovea palatinae as the posterior reference, to guide precise sectioning. Each region was assessed at three points: right ridge crest, left ridge crest, and mid-palate, resulting in nine measurement points per cast (Figure 2A).

The use of these reference points ensured reproducible sectioning across all samples, minimizing positional variability during stereomicroscopic measurements.

All the samples were stored at room temperature for 24 h after fabrication and sectioning prior to adaptation measurements. This evaluation period was selected to allow for material stabilization and minimize the residual stress or dimensional changes prior to adaptation measurements, following standard protocols for in vitro dental material studies [1]. The discrepancy between the internal surface of the record base and the external surface of the cast was measured at these points using a stereomicroscope (Leica M205 C, Leica Microsystems, Wetzlar, Germany) at 20x magnification, capable of detecting differences as small as 0.001 mm (Figures 2B, 2C). To minimize bias, the measurements were recorded by a trained technician who was blinded to the study groups.

To ensure measurement precision, the stereomicroscope was calibrated before each measurement session using a certified standard reference scale (0.01 mm precision, Leica Microsystems) to verify accuracy. Calibration checks were performed prior to every session to confirm the instrument’s precision, ensuring consistent measurement of discrepancies as small as 0.001 mm. Intra-examiner reliability was assessed by having the same technician repeat the measurements on 10 randomly selected samples after a one-week interval, with an intra-class correlation coefficient (ICC) calculated to confirm consistency (target ICC ≥0.8).

Statistical analysis

Data were analyzed using the IBM SPSS Statistics for Windows, Version 20 (Released 2011; IBM Corp., Armonk, New York, United States). Data normality was confirmed using the Shapiro-Wilk test (p>0.05) to ensure the suitability of the parametric test. The study compared the adaptation accuracy of VLC and thermoplastic denture materials using an independent t-test for intergroup analysis and one-way analysis of variance (ANOVA) with a post-hoc Bonferroni test for intragroup comparisons. Statistical significance was set at p<0.05.

## Results

Intragroup comparison of adaptation accuracy across different locations revealed significant differences between the groups (p=0.001). In the VLC group, the mean adaptation accuracy was highest in the molar region, followed by the posterior mid-palatal region and the canine region. Similarly, in the thermoplastic group, the molar region showed the highest accuracy, while the canine region and posterior mid-palatal region exhibited lower values. These findings indicate that adaptation accuracy is influenced by the anatomical region, with molar regions generally exhibiting better adaptation than canine and posterior mid-palatal regions, irrespective of the material used (Table 1).

**Table 1:** Intragroup comparison of adaptation accuracy in microns at multiple locations using one-way analysis of variance (ANOVA) Values are presented as mean ± standard deviation (SD) along with 95% confidence intervals (CI). *p<0.05 indicates statistically significant differences in adaptation accuracy among the regions within each group, n denotes the number of samples in each group.

Groups	n	Subgroups	Mean ± SD (in µm)	CI at 95%		F-stat	p-value
				Lower	Upper		
Visible light-cured (VLC) resin	20	Canine region	370.37 ± 18.05	320.71	420.04	31.32	0.001*
		Molar region	690.78 ± 27.37	620.71	760.85		
		Posterior mid-palatal region	630.85 ± 25.21	570.34	700.36		
Thermoplastic resin	20	Canine region	330.81 ± 34.45	240.91	420.71	18.43	0.001*
		Molar region	640.32 ± 42.26	530.40	750.24		
		Posterior mid-palatal region	310.79 ± 16.47	270.53	360.04		

The post-hoc Bonferroni test revealed significant differences in adaptation accuracy between the anatomical regions for both materials. In the VLC group, the canine region showed significantly lower accuracy than the molar (p=0.001) and posterior mid-palatal regions (p=0.001), whereas no significant difference was found between the molar and posterior mid-palatal regions (p=0.056). In contrast, the thermoplastic group exhibited a significantly lower accuracy in the canine region than in the molar region; however, no difference was observed between the canine and posterior mid-palatal regions (p=0.631). The molar region had significantly higher accuracy than the posterior mid-palatal region (p=0.001). These results suggest that the adaptation accuracy varies significantly by location, with the canine region generally performing worse with VLC materials, whereas thermoplastic materials show greater discrepancies between the molar and posterior mid-palatal regions (Table 2).

**Table 2:** Post-hoc Bonferroni pairwise comparisons of adaptation accuracy between the anatomical regions within each material group *p<0.05 denotes statistical significance. **The table displays the mean differences in adaptation accuracy (in microns as µm) between region pairs within each group.

Pairwise comparison		Visible light-cured resin			Thermoplastic resin		
		Mean difference**	t-stats	p-value	Mean difference**	t-stats	p-value
Canine region	Molar region	-32.41	10.52	0.001*	-30.51	7.19	0.001*
Canine region	Posterior mid-palatal region	-26.48	8.59	0.001*	2.02	0.48	0.631
Molar region	Posterior mid-palatal region	5.93	1.92	0.056	32.53	7.66	0.001*

Intergroup comparison using an independent t-test revealed a statistically significant difference in adaptation accuracy between the VLC and thermoplastic groups (p=0.001). The VLC group demonstrated higher mean accuracy (570.05 ± 27.63 µm) than the thermoplastic group (430.32 ± 35.94 µm). This suggests that the choice of material significantly influences the adaptation performance, with light-cured resins being more precise (Table 3).

**Table 3:** Intergroup comparison of overall adaptation accuracy between visible light-cured and thermoplastic resin groups using an independent t-test *p<0.05 denotes statistical significance, CI: confidence interval. **The table displays the mean differences in adaptation accuracy (in microns as µm) between groups. Data are presented as mean and standard deviation (SD), where n denotes the number of samples in each group.

Group	n	Mean ± SD (in µm)	Mean difference (in µm)**	CI at 95%		t-stat	p-value
				Lower	Upper		
Visible light-cured resin	20	570.05 ± 27.63	130.73	70.08	200.37	4.06	0.001*
Thermoplastic resin	20	430.32 ± 35.94					

## Discussion

Adaptation accuracy is a critical determinant of denture success, directly influencing retention, stability, and overall comfort [12]. In the present study, the adaptation accuracy of two widely used denture base materials, VLC resin and hard thermoplastic resin, was evaluated across key maxillary anatomical regions, including the canine, molar, and posterior palatal areas. The findings indicated that both materials demonstrated varying adaptation capabilities depending on the anatomical region, with thermoplastic resin generally offering superior adaptation, particularly in the posterior palatal region.

According to the current prosthodontic literature, an acceptable discrepancy between the internal surface of the denture base and the cast ranges from 100 to 300 µm, with optimal adaptation falling between 20 and 100 µm [12]. Our results showed that the mean discrepancy of the VLC resins was 570.05 µm, whereas that of the thermoplastic resins was significantly lower at 430.32 µm, placing both materials above the clinically acceptable range. Moreover, in the VLC group, the canine region demonstrated the least discrepancy, followed by the posterior mid-palatal and molar regions. This pattern may be attributed to the anatomical simplicity and light accessibility of the anterior region, which allows for more consistent polymerization to occur. The reliance of VLC materials on photoinitiated polymerization means that in deeper areas, such as the posterior palate, light may not penetrate effectively, resulting in under-polymerized zones and the associated shrinkage or warpage [1]. Owing to the shape of the palatal concavity, shrinkage occurs toward the residual ridge, leading to lifting of the record base in the mid-palatal region more than in the canine region, as documented by Oh et al. [13]. Consani et al. [14] suggested that the molar region is the most important site for gap space production between the palatal zone and the record base due to linear shrinkage. According to Tan et al. [15], finger pressure is insufficient for adapting the VLC denture base material.

In contrast, thermoplastic resins are not reliant on polymerization but instead on heat-softening and vacuum adaptation, offering more predictable behavior in terms of flow and conformation [1]. In our study, the posterior mid-palatal region exhibited the highest adaptation accuracy in the thermoplastic group, followed by the canine and molar regions. This reverse trend compared to the VLC group may be attributed to the molding characteristics of thermoplastics, which allow the material to be pressed into deeper areas under a consistent vacuum pressure [16].

Moreover, our intergroup comparison confirmed statistically significant differences in adaptation between VLC and thermoplastic resins, but only in the posterior palatal region. Owing to its depth and curvature, this region often presents adaptation challenges for many denture base materials [14,15]. Thermoplastics performed significantly better in this area, likely owing to their pliability during molding and lack of dependence on light for curing. Wada et al. [16] and Jagger et al. [17] found that thermoplastic materials showed an enhanced fit in the palatal regions, reinforcing the importance of pressure-based adaptation in anatomically complex zones.

The literature consistently notes that the superior adaptation of thermoplastic materials may also be influenced by their inherent properties, including their high molecular weight, thermal plasticity, and stress relaxation characteristics [1,4,11]. Ucar et al. [18] observed that thermoplastics had initial adaptation gaps between 50 and 100 µm, which although stable at first, tended to increase after 24 h due to material creep, and the polyamide denture base material exhibited a lower modulus of elasticity compared to PMMA. Similarly, Fueki et al. [19] demonstrated that thermoplastic resins maintained their shape in the short term but were subjected to long-term deformation. The 24 h assessment interval of our investigation may have resulted in an augmented discrepancy attributable to material creep in both groups, producing elevated measurements that surpass the minimum acceptable threshold of 300 µm [12].

In contrast, VLC resins, which demonstrate good early adaptation, undergo minor polymerization shrinkage post-curing [20]. Boberick et al. [21] reported a mean gap of 300-400 µm at the posterior border after one hour and seven days. The techniques used for fabrication also play a critical role. The VLC resin sheets used in this study were adapted using finger pressure and cured under controlled light exposure. Although this method minimizes air entrapment, it relies heavily on the operator’s ability to ensure consistent pressure and coverage [15]. Conversely, the thermoplastic group was adapted using a vacuum-forming machine to ensure uniform pressure and enhanced anatomical conformity. These methodological differences may partially explain the consistent superiority of thermoplastic adaptation observed in the present study.

Clinically, adaptation accuracy is particularly important in the posterior palatal seal area because poor adaptation in this region can significantly affect denture retention and sealing. Thus, our findings suggest that thermoplastic resins may be more suitable for patients requiring optimal palatal adaptation or complex posterior anatomy. Nonetheless, despite disadvantages, VLC resins remain advantageous because of their ease of use, quick polymerization, and consistent performance in anterior regions [22].

Several strategies have been explored in the literature to minimize these adaptation errors. Dual-curing protocols, step-light exposure, and stress-relieving liners have shown promise in reducing polymerization stress and improving fit [8,9,15,23]. The importance of controlled cooling and post-forming stabilization to prevent warping and improve the long-term dimensional stability of thermoplastics has been suggested in previous studies [17,19,24]. Mishra et al. [25] advocated the multistep fabrication of denture bases with three curing cycles using a VLC denture base material.

However, several limitations must be acknowledged to contextualize these results and guide future research. First, the sectioning process using a precision cutting machine introduced fine particulate debris, which, despite cleaning efforts, may have impacted the accuracy of stereomicroscopic measurements. Second, reliance solely on stereomicroscopy for measuring adaptation discrepancies may have limited the precision and comprehensiveness of the data, as this method is susceptible to operator variability and two-dimensional limitations. To enhance measurement accuracy in future studies, complementary imaging methods, such as 3D scanning (using a high-resolution intraoral scanner like the 3Shape TRIOS, 3Shape, Copenhagen, Denmark), could be employed to verify stereomicroscopic measurements and provide a more detailed, three-dimensional assessment of adaptation accuracy across the denture base surface. Third, the in vitro nature of the study limited the simulation of intraoral variables, such as mucosal compressibility, salivary effects, and thermal fluctuations. Although the light-curing parameters were standardized, future studies should investigate the impact of different units and protocols on VLC adaptation.

## Conclusions

Within the limitations of this in vitro study, it can be concluded that thermoplastic denture base resins exhibited significantly superior adaptation accuracy compared to VLC resins, particularly in the posterior mid-palatal region, where accurate tissue contact is critical for retention and stability of the prosthesis. While both materials demonstrated clinically acceptable adaptation in the anterior regions, the performance of thermoplastics in complex anatomical areas highlights their potential as more reliable materials for achieving an optimal denture fit. These findings support the selection of thermoplastic resins for cases requiring enhanced palatal adaptation and suggest the need for further research to improve polymerization control in VLC systems for use in deeper anatomical areas.
